# Retrospective cohort study of a tapered implant with high primary stability in patients with local and systemic risk factors—7-year data

**DOI:** 10.1186/s40729-018-0151-5

**Published:** 2018-12-17

**Authors:** Sotirios Konstantinos Saridakis, Wilfried Wagner, Robert Noelken

**Affiliations:** 1 Department of Oral and Maxillofacial Surgery – Plastic Surgery, University Medical Center, Johannes Gutenberg University of Mainz, Augustusplatz 2, 55131 Mainz, Germany; 2 Private Practice for Oral Surgery, Lindau/Lake Constance, Germany

**Keywords:** Local risk factors, Systemic risk factors, Immediate implant placement, Immediate provisionalization, Long-term results, Implant design, Primary stability

## Abstract

**Objectives:**

This retrospective study examined the mid- to long-term clinical and radiographic performance of a tapered implant in various treatment protocols in patients with local and systemic risk factors (RFs).

**Material and methods:**

Two hundred seven NobelActive implants were inserted in 98 patients in the period from 10/2008 to 02/2015. The subdivision of the cohort was defined by local (*n* = 40), systemic (*n* = 6), local and systemic (*n* = 8), or without any RFs (*n* = 44) to analyze implant survival and marginal bone levels.

**Results:**

Fifteen implants failed within the follow-up period. The mean follow-up period of the remaining implants was 34 months (range 12 to 77 months). The cumulative survival rate according to Kaplan-Meier was 91.5%. The survival rate for 93 implants in 45 patients with no RFs was 94.8% whereas it was 94% for 83 implants in 48 patients with local RFs (*p* = 0.618), 81.3% for 14 implants in 6 patients with systemic RFs (*p* = 0.173), and 76.5% for 17 implants in 6 patients with local and systemic risk factors (*p* = 0.006). The interproximal marginal bone level was − 0.49 ± 0.83 mm at the mesial aspect and − 0.51 ± 0.82 mm at the distal aspect in relation to implant shoulder level and showed no relevant difference in the various risk factor groups.

**Conclusions:**

It can be assumed that the negative effects of the local or/and systemic risk factors were partially compensated by the primary stability and grade of osseointegration of the NobelActive implant.

**Clinical relevance:**

The use of this system in patients with risk factors and immediate loading procedures.

## Introduction

Based on published demographic data, the median age of the world population constantly increases [1]. This has led to an increase in the number of dental implants inserted in senior individuals with local and systemic risk factors [2]. Nevertheless, despite numerous studies having been conducted on this topic, the results remain controversial, almost 50 years after the first dental implant placement, and there remains no consensus regarding the factors influencing success rates in implant dentistry.

In the presence of diabetes, there is a delayed wound healing [3], especially in patients with poor glycemic control [4]. In both experimental [5] and clinical [6] studies, a reduced osseointegration was noticed, which may have led to an increased risk of failure. Other studies [7] have alluded to the impact of radiation exposure and its side effects like xerostomia and mucositis, which may have also increased the risk for implant failure. It is believed that the irradiated hypocellular, hypovascular, and hypoxic tissues are the main cause of failures in dental implant osseointegration. Moreover, in patients who are undergoing or who earlier received treatment with cortisone or chemotherapy, implant placement might lead to loss of osseointegration [8].

In addition to systemic factors, there are also several local factors that may affect implant survival. Any factor or condition that may potentially lead to loss of primary stability should be considered as a risk for implant survival and must be detected and treated early. Such factors are the presence of severe, untreated periodontitis [9, 10], prior endodontic [11] or implant treatment at the placement site [12], previous trauma [13], alveolar clefts [14], and any other factors causing large bony defects.

Despite the existing risk factors, dental implants continue to gain popularity, and in recent years, there is an increasing demand for immediate loading and provisionalization combined with high esthetic expectations. There are several techniques and systems developed, and it can be considered that implant survival rates, although they are directly correlated to variable biological factors, approach those of traditional techniques [15]. The overall treatment time is reduced, which generally increases patient satisfaction [16].

Primary stability is a prerequisite for successful osseointegration and remains the most significant factor for the survival of dental implants [16]. Therefore, current research focuses on amelioration of existing augmentation techniques and materials or on the development of new implants with self-tapping properties for improving bone contact as well for increasing primary stability.

The aim of this clinical study was to evaluate implant survival and marginal bone level in patients with or without local and/or systemic risk factors treated with NobelActive (Nobel Biocare, Zurich, Switzerland) implants after a 2- to 7-year follow-up.

## Material and methods

### Patients

One hundred and ten patients were invited for follow-up evaluation. All patients were treated in the period from 10/2008 to 02/2015 in the Clinic of Oral and Maxillofacial Surgery, University Medical Center Mainz. Inclusion criteria were as follows: implant placement of a NobelActive implant, study subjects over 18 years old, residual bone dimension in the edentulous region of at least 5 mm in height and 4 mm in width, placement torque of at least 35 Ncm, non-smokers, and a follow-up period of least 1 year after implant placement. Exclusion criterion was treatment with bisphosphonates.

From a total of 110 invited patients, 98 patients showed up for a follow-up evaluation of the clinical and radiological status of the implants and fulfilled the abovementioned criteria. Twelve patients were dropped out because of sudden death (*n* = 1), moved to another place (*n* = 4) and were incompliant and did not appear for the follow-up (*n* = 7). The rest 98 patients included into the study were stratified into different groups according to risk factors (RFs) (local, systematic, both of them, or without risk factor), implant region, implant diameter, implant length, time of implant placement, and time of implant restoration.

Systematic risk factors included poorly controlled diabetes (HbA1c > 7%), irradiation (range; 58–64 Gy), chemotherapy (“EURO-E.W.I.N.G.99”—consisted of vincristin, ifosfamid, doxorubicin, and etoposid in one patient after resection of a sarcoma in the upper jaw), long-term therapy with corticosteroids (≥ 7.5 mg of prednisone equivalent per day during at least 90 days consecutive) [17, 18], and presence of Marfan syndrome. Local risk factors included history of severe [19, 20] periodontitis (clinical attachment loss > 5 mm)—which was not active at the time of implant placement—unsuccessful endodontic treatment with periapical pathology, implant placement in cases with moderate or severe bone defects (> 4 mm need for augmentation according to the Cologne Classification of alveolar ridge defects [21]), and replacement after removal of a previous implant (Table 1). Other co-existent diseases that do not influence the implant survival were not considered systemic risk factors.

**Table 1:** Distribution of patients with local and systemic risk factors

Systemic RF	No. of implants (*n* = 31)	No. of patients (*n* = 14)	Local RF	No. of implants (*n* = 100)	No. of patients (*n* = 54)
Diabetes	8	5	Infection	30	15
Radio(chemo) therapy	17	6	Bone defect	49	14
Cortisone medication	2	2	Implant removal	31	25
Marfan syndrome	4	1			
*Total*	*31*	*14*		*100*	54

These patients received a total of 207 NobelActive implants (Nobel Biocare, Zurich, Switzerland) placed by two experienced surgeons. Between November 2011 and February 2015, 188 implants were placed in the maxilla, and 19 implants in the mandible. All implant placement procedures were conducted at the Department of Oral and Maxillofacial Surgery of the University Medical Center, Mainz, Germany. Fifty-four implants were placed immediately after extraction, and 153 implants were placed after osseous consolidation of the extraction sockets.

Additional simultaneous bone grafting procedures, all of which were done using autologous bone, were required at 113 implant sites; another 24 sites required soft tissue augmentation with subepithelial connective tissue grafts.

Sixty-five implants were provisionalized immediately. After a healing time of at least 3 months, the final restoration was delivered. One hundred thirty implants received single-tooth restoration with a crown, 26 were loaded by an implant bridge, 19 were loaded by an implant bridge with a distal cantilever, and 32 implants were used to anchor a removable denture.

The reason for tooth removal was an endodontic failure in 38, a perio-endodontic lesion in 16, a large cyst in 11, a trauma in 24, and severe periodontitis in 56 sites. In 31 sites, a failed implant had to be removed, and in 31 sites, a pronounced bone defect was present.

The study type is a solely retrospective analysis of data obtained during follow-up in a cohort of patients treated with a CE-certified implant in a University Medical Center. Since the product is already approved in accordance with the German Medical Devices Act, additional ethics approval was not required for treatment. The study was conducted according to the principles stated in the Helsinki Declaration. Informed consent was obtained from the patient prior to any examination that was carried out for study purposes.

### Surgical technique and restoration

The cornerstones of the surgical procedure were:

- Preservation of all alveolar socket walls via longitudinal extraction after periotomy avoiding oro-vestibular luxation.
- Meticulous cleaning of the extraction site.
- Placement of rather long implants that allow for a high level of primary stability.
- Implant dimensions were as follows: implant length 8.5 mm, 24 implants; 10 mm, 6 implants; 11.5 mm, 64 implants; 13 mm, 80 implants; 15 mm, 31 implants; and 18 mm, 2 implants. Implant diameters were as follows: 3.0 mm, 25 implants; 3.5 mm, 84 implants; 4.3 mm, 90 implants; and 5 mm, 8 implants.
- If required, simultaneous reconstruction of the facial bony lamella via autologous bone chips harvested at the mandibular ramus.
- Immediate restoration by temporary crown or bridgework either by individual chairside contouring and adjustment of acrylic resin denture teeth or by lab-fabricated restorations (in case of multiple teeth); all provisional restorations were delivered on the day of implant placement and adjusted to clear all contacts in centric occlusion and during eccentric movements.
- Final restoration was delivered after 3 to 6 months.

### Follow-up and definition of outcome variables

Patients were examined clinically and radiographically at the time of implant placement and at least 12 months after implant placement. The primary outcome variable was the implant survival rate.

The secondary outcome parameter was the marginal bone level, which was determined using digital sequential periapical radiographs (XIOS XG Supreme, Dentsply Sirona, Bensheim). To ensure reproducibility between the examinations, radiographs were taken with paralleling technique using commercially available film holders (Dentsply/Rinn, Elgin, IL, USA). Specifically, the vertical distance between the implant shoulder and the bone level (mesial and distal) at the implant was measured. The distance was recorded to the nearest 0.1 mm using × 7 magnification. Attachment levels apical to the implant shoulder were designated as negative values.

### Statistical analysis

Survival probabilities were estimated by the Kaplan-Meier method on a “per implant” basis [14]. The endpoint of interest was implant failure. To compare the survival distribution of two samples (no RF, local RF, systemic RF, local and systemic RF; maxilla vs. mandible; different implant diameters and lengths; immediate vs. delayed placement; immediate restoration and immediate provisionalization vs. other treatment concepts; with or without bone grafting), the log-rank test was used.

Subpopulations within the study group (immediate vs. delayed placement) were compared using the Wilcoxon-Mann-Whitney non-parametric *U* test. The reported *p* values were two sided. All calculations were carried out using SPSS for Mac, Version 22 (SPSS Inc., Chicago, IL, USA).

## Results

Ninety-eight patients with 207 implants complied with the treatment protocol attended the follow-up.

### Implant survival

During the follow-up period, 15 implants failed in 12 patients. Age and gender were not correlated with a lower implant survival. The implant losses occurred in a time range between 0.5 and 39 months following implant placement (mean 7.3 ± 11.1 months). The reasons for implant failure were loss of osseointegration (*n* = 11), peri-implantitis (*n* = 2), occlusal trauma (*n* = 1), and mechanical complication (*n* = 1). From these 15 failures, 6 implants failed after delivery of the final prosthetic restoration while 9 implants failed without being prosthetically restored. The majority of failures (*n* = 13) occurred during the first year after placement (Table 2).

**Table 2:** Clinical parameters and reason for implant failures of 15 implants in 12 patients

Region	Systemic RF	Local RF	Immediate procedure	Bone grafting	Time of failure (months)	Reason
26	No	Periodontitis	Immediate restoration	No	5	Screw fracture
15	Marfan syndrome	No	No	Yes	39	Peri-implantitis
14	No	No	No	No	1	Loss of osseointegration
22	No	Defect	Immediate placement	Yes	3	Loss of osseointegration
15	No	Former impl. removal	Immediate placement	Yes	10	Loss of osseointegration
22	No	Defect	No	Yes	3	Loss of osseointegration
24	No	Periodontitis	Immediate placement/restoration	Yes	28	Peri-implantitis
12	Radiochemotherapy	Defect	No	Yes	3	Loss of osseointegration
13	Radiochemotherapy	Defect	No	Yes	3	Loss of osseointegration
22	Radiochemotherapy	Defect	No	Yes	3	Loss of osseointegration
23	Radiochemotherapy	Defect	No	Yes	3	Loss of osseointegration
23	No	No	Immediate placement/restoration	Yes	4	Loss of osseointegration
22	Diabetes	No	Immediate restoration	No	1	Occlusal trauma
12	No	Defect	Immediate placement	Yes	1	Loss of osseointegration
32	No	No	No	No	1	Loss of osseointegration

**Fig. 1:**
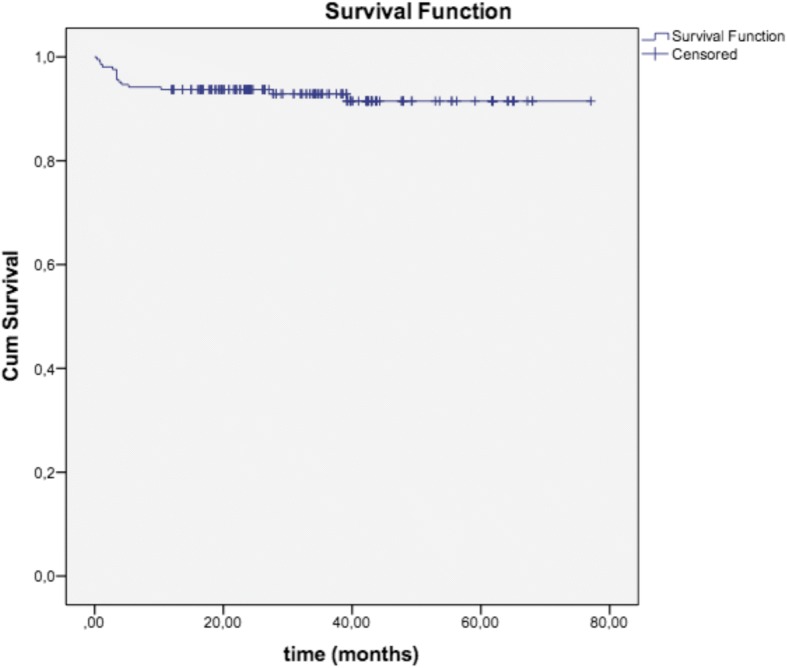
On Kaplan-Meier survival estimates, the cumulative survival rate was 91.5%

Cumulative survival rates (SRs) were 91.5% for all implants (Fig. 1). The remaining 194 implants in 88 patients were evaluated 12 to 77 months (mean 33.9 ± 14.7 months) following implant placement. Two more implants in 2 patients failed in this period.

**Fig. 2:**
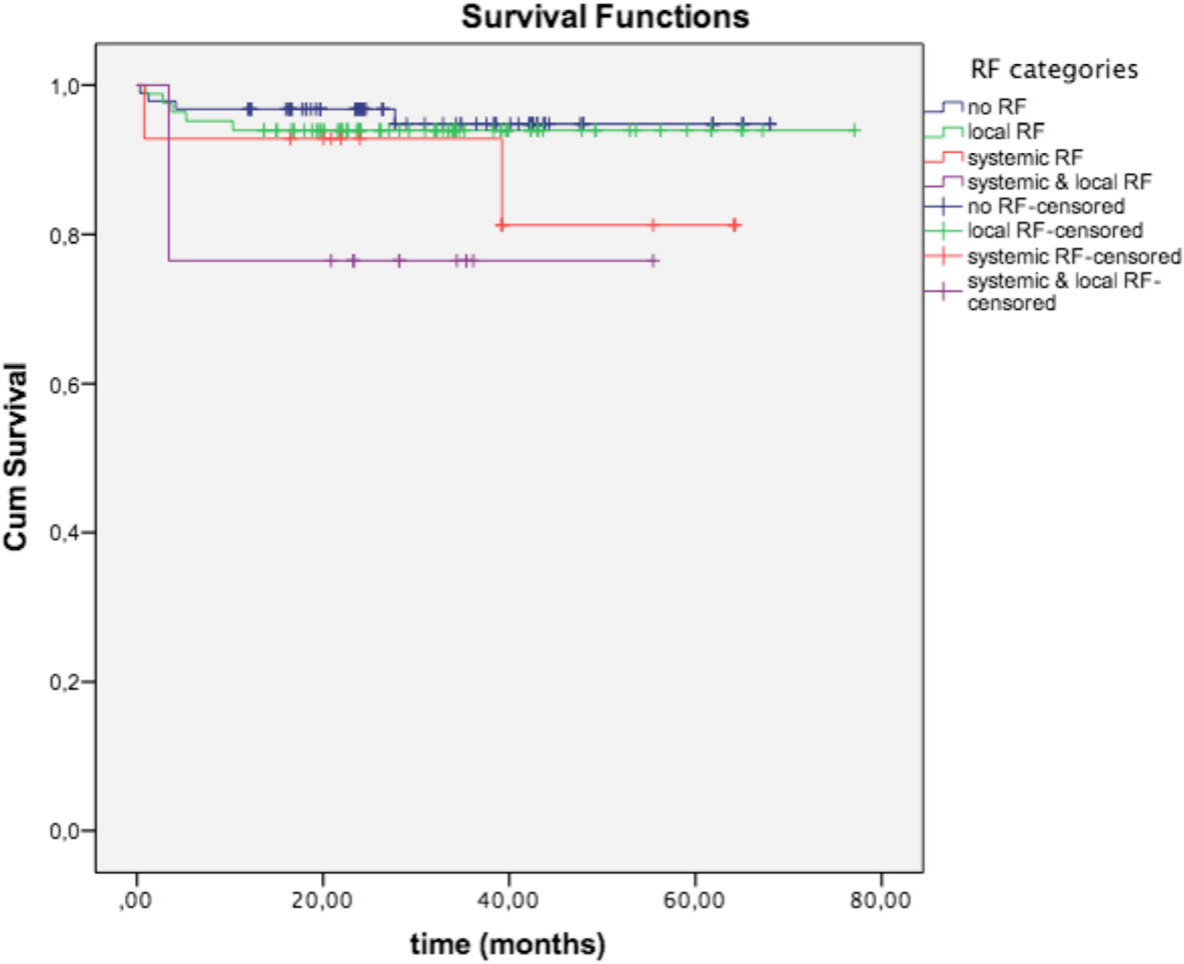
The cumulative survival rates for the different RF groups revealing a significant difference for patients without any and patients with local and systemic RFs (log rank, *p* = 0.006)

The survival rate for 93 implants in 45 patients with no RFs was 94.8% whereas it was 94% for 83 implants in 48 patients with only local RFs (log rank, *p* = 0.618), 81.3% for 14 implants in 6 patients with only systemic RFs (*p* = 0,173), and 76.5% for 17 implants in 6 patients with both local and systemic risk factors (*p* = 0.006) (Fig. 2). The survival rate of implants in patients with no RFs compared to those with local and systemic RFs displayed a significant difference.

The implant survival was 91.3% in maxilla (*n* = 188) while it was 94.7% in mandible (*n* = 19) (log rank, *p* = 0.788). One hundred twenty-two implants were placed in the anterior region, 65 in the premolar region, and 20 in the molar region. From the total of 15 implant removals (SR 92.7%), 11 (SR 90.98%) occurred in the anterior region, 3 (SR 95.38%) in the premolar region, and 1 (SR 95.0%) in the molar region, so no significant difference was found regarding implant survival rate between the regions (log rank, *p* = 0.478). The implant diameter and length did not show any difference in survival rate. Delayed implant placement (*n* = 153, 91.9%) did not reveal a significant higher survival rate than implants placed immediately into extraction sites (*n* = 54, 90%) (*p* = 0.603). Delayed loaded implants (*n* = 142, 93%) did not show a significant higher survival rate than immediately provisionalized implants (*n* = 65, 89.3%) (*p* = 0.935). The treatment concept of immediate implant placement and immediate provisionalization (IPP, *n* = 30) did not have a negative impact on implant survival (IPP 92.1%, all other implants (*n* = 177) 91.4%, *p* = 0.809). Although the implant survival was lower in the group of implants with simultaneous bone grafting (*n* = 113, 88.1%) compared to those without (*n* = 94, 95.7%), the difference did not reach the level of significance (*p* = 0.151).

### Marginal bone levels

Regarding the implant shoulder level, the average interproximal marginal bone level was − 0.49 ± 0.83 mm (range, 0 to − 3.3 mm) at the mesial aspect and − 0.51 ± 0.82 mm (range, 0 to − 3.9 mm) at the distal aspect of the implants.

When the marginal bone level was considered as a function of time, there was no strict correlation between the marginal bone status and the length of the follow-up period (*r* = 0.025, *p* = 0.764; Spearman rank correlation coefficient), suggesting that bone levels remained, by and large, stable during the observation period.

No relevant difference of marginal bone level was noticed for implants in the different risk factor groups (no RF − 0.53 ± 0.82, local RF − 0.50 ± 0.67, systemic RF − 0.63 ± 0.64, local and systemic RF − 0.17 ± 0.23).

The marginal bone level for implants inserted immediately (− 0.46 ± 0.68 mm) or delayed (− 0.52 ± 0.75 mm) did not show a difference (*p* = 0.676).

## Discussion

Most implant studies deal only with local risk factors, although the existence of systemic risk factors plays a significant role to the implant survival. We use the NobelActive dental implant in this study in order to investigate if this promising implant with the special design could achieve better survival rates in difficult situations with several risk factors. The present study revealed no statistically significant difference in the survival rate of implants between patients with systemic RFs and healthy controls. The only statistically significant difference concerned implants of patients with both systemic and local RFs (*p* = 0.006).

Despite the heterogeneity of the studies, it appears that diabetes (both types) [22, 23] is related to delayed wound healing [24], alterations in early bone healing due to poor glycemic control [25], and marginal bone loss [26, 27]. Moreover, greater failure rates were found in patients who had diabetes for longer time periods [28]. Other studies, however, did not find a difference in survival rates between diabetics and healthy patients [29, 30].

Our study included a patient after resection of an Ewing sarcoma in the left maxilla who was treated with dental implants 2 years after the hemimaxillectomy and 1 year after the completion of the combined radiochemotherapy in order to achieve an oral rehabilitation. 3 months after the insertion of six implants; four of them should be removed because of lack of osseointegration. At the moment of the implant placement as well as at the moment of the implant removals, there was no recurrence of the sarcoma. Studies comparing patients that had undergone radiation treatments and non-irradiated controls have found similarly ambiguous results [31]. Ihde et al. [32] exhibited a two to three times greater failure rate in irradiated bone compared to non-irradiated. Curi et al. showed that mode of radiation therapy delivery (*p* = 0.005) had a statistically significant influence on implant survival [33].

In one patient with three implants and long intake of corticosteroids against rheumatoid arthritis (7.5 mg prednisolone per day for at least 2 years), no complication was detected in our study. Long-term use of corticosteroids can also lead to implant failures, according to Wood and Vermilyea [34] by modifying the patient’s response to bacterial infection [35], but at the present time, there is no important consideration for avoiding implant placement in those patients [36].

One implant removal was also performed on a patient with Marfan syndrome. While there are no reports in the literature regarding implant placement in patients with Marfan syndrome [37], analytical studies of bone mineral density reported adult patients demonstrated a high risk for developing osteoporosis [38]. Implant failures in postmenopausal women with osteoporosis [39] can lead to an assumption that this category of patients might be in higher risk for implant loss.

Our study showed no statistically significant difference between groups with local RFs such as periodontitis or implant placement in areas with a previous endodontic treatment and without any RFs. Hultin et al. [40] reported that a local specific inflammatory reaction related to the presence of bacteria occurs around the implants leading to marginal bone loss and infection (peri-implantitis) and that its incidence is four times higher in patients with history of chronic periodontitis than in patients who have never manifested periodontal disease. Moreover, patients without history of periodontitis present an average of 96.5% of implant survival in comparison to 90.5% of survival in individuals with history of periodontitis [40, 41]. Similar findings [42] were observed by the development of a retrograde peri-implantitis and how it affects the implant either from an adjacent tooth or from remaining infected periapical tissue in the prior position of the tooth [13]. The incidence of retrograde peri-implantitis was 7.8% in a study of 128 implants placed in areas adjacent to the teeth that had received endodontic treatment [43]. The mechanism of this procedure is considered multifaceted [15].

The survival rate for 93 implants in 45 patients with no RFs was 94.8%. Moraschini et al. [44] have exhibited in their systematic review, based on 7711 implants, similar SRs with cumulative mean values of 94.6%. Moreover, in the subcohort of our study, by 44 of the above 93, implants were performed immediate procedures (25 immediate implantations, 35 immediate restorations, 16 both of them) so the survival rate could be also correlated with studies tasking with immediate procedures as well [45–47].

Very encouraging results came out by a secondary implant placement in areas of previous removed implants. Only one implant was lost out of a total of 31, which translates to a survival rate of 96.8%. According to studies by Grossmann and Levin [12] and Greenstein and Cavallaro [48], survival rates ranged between 50 and 100%.

The timing of implant placement and prosthetic restoration showed no statistically significant influence in our study. A meta-analysis of Hartog et al. [41] showed a survival rate of 95.5% and no difference between immediate, immediate-delayed, and delayed implant placement in the esthetic zone. Schropp et al. [49] used CBCT technology to test the remaining vestibular bone in conventional implant placements and immediate procedures and came to no statistically significant differences regarding bone preservation. Esposito et al. [46] demonstrated an increased risk of implant failure in patients after immediate implant placement (9%) when compared to delayed implant placement (2%). In our study, we found no significant difference between delayed implant placement (*n* = 153, 91.9%) and immediate implant placement (*n* = 54, 90%) (*p* = 0.603). Finally, Rocci et al. [47] presented the results of a study looking at a 9-year follow-up of Brånemark implants with a survival rate of immediately loaded implants of 92.2% that was very similar to the results of this study.

We found no difference of survival rate between implants with and without augmentation procedures. The relative lower implant survival rate of implants with augmentation procedures (*n* = 113, 88.1%) compared to those without (*n* = 94, 95.7%) can be explained from the fact that the sites needed augmentation showed a significant bone loss that maybe cannot be compensated from the augmentation procedure.

A major weakness of the study is the relative small size of the group with systemic RFs (14 implants by 6 patients) as well as of the group with systematic and local RFs (17 implants by 6 patients). Because of the relative small size of the sample, this study can provide the basis for further investigations based on larger patient samples.

## Conclusions

This study was based on the recruitment of a quite heterogeneous group of patients treated with NobelActive implants, for the purpose of investigating the influence of local and systemic risk factors on implant survival and marginal bone levels. It can be considered that the presence of local or systemic risk factors does not influence implant survival whereas the combination of local and systemic risk factors reveals significantly lower implant survival.
